# Association between folic acid use during pregnancy and gestational diabetes mellitus: Two population-based Nordic cohort studies

**DOI:** 10.1371/journal.pone.0272046

**Published:** 2022-08-11

**Authors:** Laura Pazzagli, Silvia Segovia Chacón, Christos Karampelias, Jacqueline M. Cohen, Gabriella Bröms, Helle Kieler, Ingvild Odsbu, Randi Selmer, Olov Andersson, Carolyn E. Cesta

**Affiliations:** 1 Department of Medicine Solna, Centre for Pharmacoepidemiology, Karolinska Institutet, Stockholm, Sweden; 2 Department of Cell and Molecular Biology, Karolinska Institutet, Stockholm, Sweden; 3 Department of Chronic Diseases, Norwegian Institute of Public Health, Oslo, Norway; 4 Department of Internal Medicine, Danderyd Hospital, Stockholm, Sweden; 5 Department for Laboratory Medicine, Karolinska Institutet, Stockholm, Sweden; 6 Department of Mental Disorders, Norwegian Institute of Public Health, Oslo, Norway; Fudan University, CHINA

## Abstract

**Introduction:**

Inconsistent results have been reported on the association between folic acid use in pregnancy and risk of GDM. The aim of this study was to estimate the association between folic acid use and GDM in two population-based Nordic cohorts.

**Material and methods:**

Two cohort studies were conducted using data from the national population registers in Norway (2005–2018, n = 791,709) and Sweden (2006–2016, n = 1,112,817). Logistic regression was used to estimate the associations between GDM and self-reported folic acid use and prescribed folic acid use, compared to non-users, adjusting for covariates. To quantify how potential unmeasured confounders may affect the estimates, E-values were reported. An exposure misclassification bias analysis was also performed.

**Results:**

In Norwegian and Swedish cohorts, adjusted odds ratios (ORs) and 95% confidence intervals (CIs) for maternal self-reported folic acid use were 1.10 (1.06–1.14) and 0.89 (0.85–0.93), with E-values of 1.43 (1.31) and 1.50 (1.36), respectively. For prescribed folic acid use, ORs were 1.33 (1.15–1.53) and 1.56 (1.41–1.74), with E-values of 1.99 (1.57) and 2.49 (2.17), in Norway and Sweden respectively.

**Conclusions:**

The slightly higher or lower odds for GDM in self-reported users of folic acid in Norway and Sweden respectively, are likely not of clinical relevance and recommendations for folic acid use in pregnancy should remain unchanged. The two Nordic cohorts showed different directions of the association between self-reported folic acid use and GDM, but based on bias analysis, exposure misclassification is an unlikely explanation since there may still be differences in prevalence of use and residual confounding. Prescribed folic acid is used by women with specific comorbidities and co-medications, which likely underlies the higher odds for GDM.

## Introduction

Gestational diabetes mellitus (GDM) is one of the most common complications of pregnancy, affecting up to 15% of pregnancies worldwide [1, 2]. While several risk factors for GDM have been identified (i.e., elevated body mass index, age, ethnicity, and family history of diabetes), there is limited knowledge on possible prevention strategies aside from lifestyle modification.

Since the late 1990’s, women in many European countries, including Norway and Sweden, have been advised to take 0.4 mg of folic acid daily from the time they plan to get pregnant to the end of the first trimester to reduce the risk of neural tube defects in their offspring. Further, for women at risk of a neural tube defects recurrence, and those who have diabetes, epilepsy, obesity, or use certain medications, it is recommended to take 4–5 mg of folic acid supplement per day [3]. Generally, folic acid is a common dietary supplement and emerging evidence has suggested that physiologically insufficient levels of folic acid intake may play a role in preventing metabolic disturbances, including insulin resistance [4], and it has been hypothesized that folic acid supplementation may then prevent the development of GDM [5].

Therefore, a number of studies have assessed the association between pre-pregnancy/early pregnancy folic acid supplementation and GDM, however, they report inconsistent results ranging from a protective role of folic acid to no effect or harmful associations [6–8]. Further, a 2021 meta-analysis reported that high maternal folate status measured in serum or red blood cells was associated with increased risk of GDM [9].

The aim of this study was to assess the association between folic acid use in early pregnancy and the development of GDM in two large population-based cohorts of pregnancies in Norway and Sweden.

## Materials and methods

### Data sources and study populations

Two cohort studies were conducted using data from the national population registers in Norway and Sweden. A unique personal identity number is issued to all residents of Norway and Sweden upon birth or immigration, and can be used to link individual-level data from the population registers including Medical Birth Registers (MBRs), Prescribed Drug Registers (PDRs), National Patient Registers (NPRs), Cause of Death Registers, and other registers containing information on other demographic factors such as education and migration.

**Fig 1:**
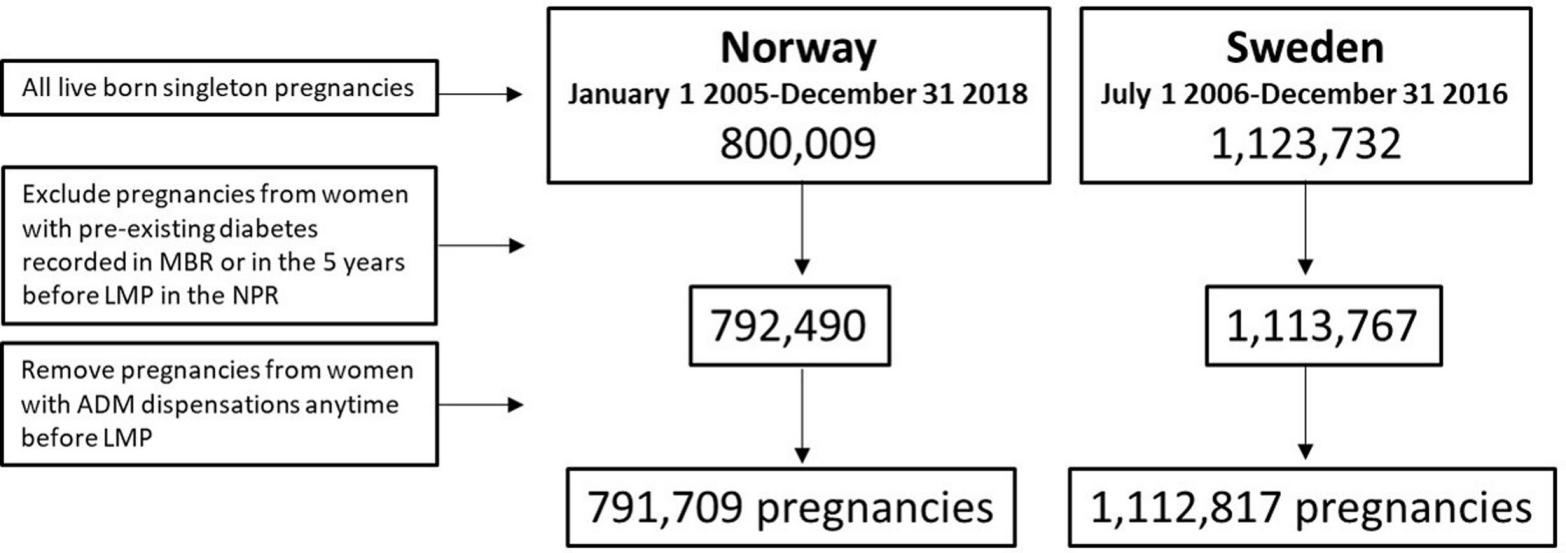
Flowchart illustrating the derivation of the study populations.

The MBRs were used to identify all pregnancies resulting in a singleton livebirth after 12 weeks gestation in Norway and from 28 weeks before 2008 or 22 weeks gestation after 2008 in Sweden. Included pregnancies were for births occurring between 1 January 2005 and 31 December 2018 (Norway) and 1 July 2006 and 31 December 2016 (Sweden). Pregnancies from women with a diagnosis of diabetes within 5 years prior to the start of pregnancy recorded in the NPRs (ICD-10 codes E10, E11, E14, O24.0, O24.1) or reported in the MBRs were excluded. In Norway, data from the NPR were available from 2008 and therefore only pregnancies ending after 2013 had the full 5-year look back period available. Further, pregnancies of women with antidiabetic medication (Anatomical Therapeutic Chemical (ATC) code A10) dispensations at any time before pregnancy as recorded in the PDRs were excluded. However, women who were dispensed metformin (ATC code A10BA02) any time prior to pregnancy with no diabetes diagnoses were not excluded, as metformin can be used as a fertility enhancing drug. A total of 791,709 pregnancies from Norway and 1,112,817 pregnancies from Sweden were included (**Fig 1**).

### Exposure: Folic acid use

Mutually exclusive folic acid user groups were identified: prescribed folic acid use, self-reported folic acid use, and no folic acid use. Women who both reported folic acid use and had a dispensing of folic acid were classified into the prescribed folic acid group.

#### Self-reported folic acid use

In Norway, at the first antenatal care visit around 10–12 weeks gestation, the pregnant woman is specifically asked if she used folic acid during pregnancy, which is recorded in a checked box (yes/no) in the patient’s chart and then included in the MBR since 1999.

In Sweden, at the first antenatal care visit around 10–12 weeks gestation, the pregnant woman is asked about any medication use during pregnancy, and the information is recorded as free text in the patient’s chart and then included in the MBR, since 1995. When possible, the free text is converted into an ATC code by the register holder. To extract the information about folic acid use from the MBR, we identified ATC codes, folic acid supplement, and pregnancy-specific multivitamin supplement names. For further details see the (S1 Methods in S1 File).

#### Dispensations of folic acid prescriptions

The PDRs includes information on all prescribed drugs dispensed for the entire Norwegian and Swedish populations since 1 January 2004 and 1 July 2005, respectively. Pregnancies with at least 1 dispensation of prescribed folic acid in the 90 days before the start of pregnancy to the end of the 1^st^ trimester of pregnancy were identified by the ATC codes B03BB01 and B03BB51. The dose of these dispensations ranged from 1 to 5 mg.

### Outcome: Gestational diabetes

GDM was identified by a variable from the MBR in Norway or ICD-code O24.4 registered in the MBRs or in the NPRs with a diagnosis date after the start of the 2^nd^ trimester of pregnancy, or by a first dispensation of an antidiabetic medication in the PDR after the start of the 2^nd^ trimester.

### Covariates

Data on covariates were collected from the MBR, the PDR, and the NPR and included the year of delivery, maternal age at delivery, parity, smoking in early pregnancy, early-pregnancy body mass index (BMI) calculated from maternal height and weight, cohabitation with partner, maternal country of birth (Nordic, Non-Nordic), and GDM in previous pregnancy (ICD-10 O24.4). Highest achieved maternal education in the year of birth was collected from registers at the national statistics agencies. Additionally, diagnoses of epilepsy, hypertension, psychiatric disorders, and other comorbidities related to folic acid use (e.g., inflammatory join diseases, psoriasis, Crohn’s disease, immune deficiency, etc) recorded in the MBRs, and dispensations in the PDRs of antiepileptics, medications used to treat psychiatric conditions, methotrexate, and glucocorticoids in the six months before the start of pregnancy were identified. ICD and ATC codes used to identify these diagnoses and medications are listed in the **S1 Table in S1 File**.

### Statistical analysis

Analyses were conducted separately on the Norwegian and Swedish cohorts. Logistic regression models using generalized estimating equations were fitted using the R package ‘drgee’ [10, 11], with robust standard errors to account for dependency in the data, since women could have more than one pregnancy. Odds ratios (ORs) and 95% confidence intervals (CIs) were estimated for the association with GDM in women with self-reported folic acid use, and women with prescribed folic acid use, compared to women with no folic acid use (reference group). Crude and adjusted models were fitted. The adjusted model included birth year, maternal age, cohabitation, smoking, maternal country of birth, diagnoses of epilepsy, hypertension, psychiatric conditions, other comorbidities related to folic acid use, and use of antiepileptics, psychiatric medication, methotrexate, or glucocorticoids.

Values for early-pregnancy BMI were missing for 50% of the Norwegian study population, therefore BMI was excluded from the main analysis models. Supplementary analyses were conducted using a missing indicator for women without available early-pregnancy BMI.

#### Bias analyses

A sensitivity analysis for unmeasured confounding was performed and E-values were provided to assess the minimum strength of association needed between an unmeasured confounder and both the exposure and outcome variables to totally explain away the exposure-outcome association. A large E-value indicates that a considerably large amount of unmeasured confounding is needed to explain away the estimated association. Conversely, a small E-value implies that only a little amount of unmeasured confounding could explain away the estimated association [12, 13].

Lastly, as the sensitivity of the self-reported folic acid use exposure in the Swedish data is likely to be low, we conducted an exposure misclassification bias analysis to provide an overview of the potential bias introduced by different levels of exposure misclassification. We calculated corrected crude ORs and 95% CIs assuming different levels of sensitivity and specificity (85–99%) for the self-reported folic acid use, using the R function ‘misclassification’ in the package “episensr” [14, 15]. Higher sensitivities indicate that more women with folic acid use are accurately classified as using folic acid, and higher specificities indicate that more women without folic acid are accurately classified as not using folic acid.

All the analyses were performed using R [16].

### Ethical approval

The study was approved by the Stockholm Regional Ethical Review Board (Dnr 2015/1826-31/2, 2017/2238-32, 2018/1790-32 and 2018/2211-32) and by the Norwegian Data Inspectorate and the Regional Ethics Committee for Medical Research of South-East Norway. In both countries, register-based studies are exempt from informed consent. Anonymized data are received by researchers from the register holders.

## Results

**Tables 1 and 2** report the characteristics of the pregnant women in the two cohorts. Of the total 791,709 pregnancies from Norway and 1,112,817 pregnancies from Sweden, 68.0% and 22.9% had self-reported folic acid use in early pregnancy, respectively, with increasing use over the study period in both countries. In both Norway and Sweden, women who self-reported folic acid use, compared to folic acid non-users, had lower parity, were less likely to smoke, more often were cohabiting with a partner, had higher education levels, and a higher proportion were born in the Nordic countries.

**Table 1:** Characteristics of study population from Norway including all pregnancies recorded in the Medical Birth Register from mothers without pre-existing diabetes or pre-pregnancy antidiabetic medication use from 2005 to 2018.

NORWAY							
n = 791,709 pregnancies							
		No Folic acid use		Self-Reported Folic acid use		Prescribed Folic acid use	
		N	%	N	%	N	%
	Total	245,815	31.0	538,981	68.0	6,913	1.0
**COVARIATES**							
Birth year *% per total pregnancies in birth year category	2005–2008	98,491	44.0*	123,160	55.0*	2,328	1.0*
	2009–2012	74,067	31.7*	157,718	67.4*	2,116	0.9*
	2013–2018	73,257	21.9*	258,103	77.3*	2,469	0.7*
Age at delivery, years	< 20	7,301	3.0	6,278	1.2	210	3.0
	20–24	40,702	16.5	64,670	12.0	1,231	17.8
	25–29	74,858	30.5	175,661	32.6	2,130	30.8
	30–34	75,831	30.8	188,715	35.0	2,051	29.7
	35–39	38,472	15.7	87,474	16.2	1,062	15.4
	40–44	8,177	3.3	15,478	2.9	212	3.1
	> = 45	474	0.2	705	0.1	17	0.2
	Missing	0	0.0	0	0.0	0	0.0
Parity	0	93,125	37.9	239,104	44.4	3,100	44.8
	1+	152,690	62.1	299,877	55.6	3,813	55.2
	Missing	0	0.0	0	0.0	0	0.0
Body Mass Index (kg/m^2^), early pregnancy	0-<18	2,561	1.0	7,062	1.4	92	1.3
	18-<25	48,124	19.6	201,286	37.3	1,814	26.2
	25-<30	19,032	7.7	67,468	12.5	723	10.5
	30-<35	7,54	3.1	24,490	4.5	323	4.7
	35-high	3,479	1.4	10,832	2.0	149	2.2
	Missing	165,115	67.2	227,843	42.3	3,812	55.1
Smoking, early pregnancy	No	156,964	63.9	453,043	84.1	5,072	73.3
	Yes	26,793	10.9	38,431	7.1	772	11.2
	Missing	62,058	25.2	47,507	8.8	1.069	15.5
Cohabitation	Not cohabiting	22,712	9.2	26,469	4.9	620	9.0
	Cohabiting	220,481	89.7	508,496	94.4	6,227	90.1
	Missing	2,622	1.1	4,016	0.7	66	1.0
Maternal birth country	Nordic	161,179	65.6	444,735	82.5	4,380	63.4
	Non-Nordic	81,665	33.2	90,035	16.7	2,469	35.7
	Missing	2,971	1.2	4,211	0.8	64	0.9
Highest achieved maternal education, year of delivery	Compulsory	61,007	24.9	71,466	13.2	1,902	27.4
	Secondary	65,431	26.6	139,560	25.9	1,988	28.8
	Post-secondary	91,045	37.0	303,832	56.4	2,307	33.4
	Missing	28,332	11.5	24,123	4.5	716	10.4
Gestational diabetes in a previous pregnancy	No	244,703	99.5	535,859	99.4	6,869	99.4
	Yes	1,112	0.5	3,122	0.6	44	0.6
Epilepsy	No	244,641	99.5	535,665	99.4	5,912	85.5
	Yes	1,174	0.5	3,316	0.6	1,001	14.5
Hypertension	No	244,616	99.5	535,010	99.4	6,876	99.5
	Yes	1,199	0.5	2,971	0.6	37	0.5
Psychiatric Disorder	No	232,983	94.8	506,742	94.0	6,362	92
	Yes	12,832	5.2	32,239	6.0	551	8.0
Other comorbidities related to folic acid use	No	243,794	99.2	532,639	98.8	6,660	96.3
	Yes	2,021	0.8	6,342	1.2	253	3.7
Antiepileptic medication	No	244,638	99.5	535,532	99.4	5,801	83.9
	Yes	1,177	0.5	3,449	0.6	1,112	16.1
Medication used to treat psychiatric conditions	No	232,572	94.6	507,766	94.2	6,174	89.3
	Yes	13,243	5.4	31,215	5.8	739	10.7
Methotrexate use	No	245,775	100.0	538,881	100.0	6,817	98.6
	Yes	40	0.0	100	0.01	96	1.4
Glucocorticoid use	No	242,997	98.9	530,885	98.5	6,613	95.7
	Yes	2,818	1.1	8,096	1.5	300	4.3

**Table 2:** Characteristics of study population from Sweden including all pregnancies from mothers without pre-existing diabetes or pre-pregnancy antidiabetic medication use from 1 July 2006 to 31 December 2016.

SWEDEN							
n = 1,112,817 pregnancies							
		No Folic acid use		Self-Reported Folic acid use		Prescribed Folic acid use	
		N	%	N	%	N	%
	Total	838,730	75.4	254,280	22.9	19,807	1.8
**COVARIATES**							
Birth year *% per total pregnancies in birth year category	2006–2008	216,854	86.2*	30,232	12.0*	4,471	1.8*
	2009–2012	323,835	76.2*	93,057	21.9*	7,940	1.9*
	2013–2016	298,041	68.3*	130,991	30.0*	7,396	1.7*
Age at delivery, years	< 20	13,392	1.6	2053	0.8	85	0.4
	20–24	114,492	13.7	27,649	10.9	1,465	7.4
	25–29	249,385	29.7	77,414	30.4	4,746	24.0
	30–34	281,216	33.5	91,560	36.0	7,203	36.4
	35–39	147,532	17.6	45,913	18.1	4,932	24.9
	40–44	31,142	3.7	9,255	3.6	1,288	6.5
	> = 45	1,571	0.2	436	0.2	88	0.4
	Missing	0	0.0	0	0.0	0	0.0
Parity	0	362,181	43.2	119,850	47.1	10,406	52.5
	1+	476,549	56.8	134,430	52.9	9,401	47.5
	Missing	0	0.0	0	0.0	0	0.0
Body Mass Index (kg/m^2^), early pregnancy	0-<18	11,297	1.4	3,326	1.4	263	1.3
	18-<25	466,329	55.6	155,178	61.0	10,123	51.1
	25-<30	194,933	23.2	60,490	23.8	5,171	26.1
	30-<35	69,189	8.2	19,924	7.8	2,093	10.6
	35-high	29,009	3.5	8,216	3.2	895	4.5
	Missing	67,973	8.1	7,146	2.8	1,262	6.4
Smoking status, early pregnancy	No	744,511	88.8	241,787	95.1	17,930	90.3
	Yes	52,252	6.2	11,540	4.5	1,179	6.0
	Missing	41,967	5.0	953	0.4	725	3.7
Cohabitation	Not cohabiting	101,287	12.1	14,488	5.7	1,878	9.5
	Cohabiting	737,443	87.9	239,792	94.3	17.929	90.5
	Missing	0	0.0	0	0.0	0	0.0
Maternal birth country	Nordic	635,273	75.7	205,022	80.6	15,415	77.8
	Non-Nordic	203,457	24.3	49,258	19.4	4,392	22.2
	Missing	0	0.0	0	0.0	0	0.0
Highest achieved maternal education, year of delivery	Compulsory	99,814	11.9	19,274	7.6	1,995	10.1
	Secondary	414,983	49.5	119,540	47.0	9,715	49.0
	Post-secondary	302,971	36.1	111,909	44.0	7,857	39.7
	Missing	20,962	2.5	3,557	1.4	240	1.2
Gestational diabetes in a previous pregnancy	No	835,484	99.6	253403	99.7	19,698	99.4
	Yes	3,246	0.4	877	0.3	109	0.6
Epilepsy	No	835,720	99.6	253,355	99.6	17,653	89.1
	Yes	3,010	0.4	925	0.4	2,154	10.9
Hypertension	No	833,074	99.3	252,935	99.5	19.545	98.7
	Yes	5,656	0.7	1345	0.5	262	1.3
Psychiatric Disorder	No	828,275	98.8	251,241	98.8	19,300	97.4
	Yes	10,455	1.2	3,039	1.2	507	2.6
Other comorbidities related to folic acid use	No	835,987	99.7	253,591	99.7	19,200	96.9
	Yes	2,743	0.3	689	0.3	607	3.1
Antiepileptic medication	No	834828	99.5	253,199	99.6	17,293	87.3
	Yes	3,902	0.5	1,081	0.4	2,514	12.7
Medication used to treat psychiatric conditions	No	780,413	93.0	237,592	93.4	17,041	86.0
	Yes	58,317	7.0	16,688	6.6	2,766	14.0
Methotrexate use	No	838,570	100.0	254,239	100.0	19,702	99.5
	Yes	160	0.0	41	0.0	105	0.5
Glucocorticoid use	No	824,949	98.4	250,203	98.4	18,723	94.5
	Yes	13,781	1.6	4,077	1.6	1,084	5.5

For 1.0% of pregnancies in Norway and 1.8% of pregnancies in Sweden, the women had filled a prescription for folic acid in the 90 days before LMP or during 1^st^ trimester, and the proportions of these pregnancies remained steady throughout the study period. **Tables 1 and 2** show that compared to folic acid non-users, women using prescription folic acid had higher early-pregnancy BMI, higher rates of diagnoses of epilepsy (Norway: 14.5% vs 0.5%; Sweden 10.9% vs 0.4%), use of antiepileptic medication (Norway: 16.1% vs 0.5%; Sweden: 12.7% vs 0.5%), psychiatric disorders diagnoses and related medications, other comorbidities related to folic acid use (Norway: 3.7% vs 0.8%; Sweden: 3.1 vs 0.3%), as well as methotrexate and glucocorticoid use.

In the Norwegian study population, the overall proportion of GDM was 3.2% and it was highest in mothers with prescribed folic acid use (4.3%) and lowest in the folic acid non-users (2.9%). In Sweden, the overall proportion of GDM was 1.2%, and it was highest in the prescribed folic acid group (2.2%) and lowest in the self-reported folic acid group (1.0%).

**Table 3** reports the ORs and 95% CIs for the associations between self-reported folic acid use in pregnancy and GDM. In the Norwegian cohort, there were slightly higher odds for GDM (adjusted OR = 1.10; 95% CI 1.06–1.14, 3.3% exposed GDM cases vs 2.9% unexposed GDM cases) and in the Swedish cohort, there were lower odds for GDM (adjusted OR = 0.89; 95% CI 0.85–0.93, 1% exposed GDM cases vs 1.3% unexposed GDM cases). In the supplementary analysis with early-pregnancy BMI adjustment, conducted using the missing indicator for pregnancies without available information on early-pregnancy BMI values (**S2 Table in S1 File**), the strength of the association between self-reported folic acid use and GDM remained similar (Norway: OR = 1.13; 95% CI 1.09–1.17; Sweden: OR = 0.92; 95% CI 0.88–0.96).

**Table 3:** Crude and adjusted odds ratios and 95% confidence intervals estimating the association between self-reported folic acid use and gestational diabetes in mothers from Norway and Sweden.

	Norway				Sweden			
	No GDM n (%)	GDM n (%)	Crude OR (95% CI)	Adjusted * OR (95% CI)	No GDM n (%)	GDM n (%)	Crude OR (95% CI)	Adjusted * OR (95% CI)
**No Folic acid use**	238,755 (97.1)	7,060 (2.9)	Reference	Reference	828,160 (98.7)	10,570 (1.3)	Reference	Reference
**Self-reported folic acid use**	520,966 (96.7)	18,015 (3.3)	1.17 (1.14, 1.20)	1.10 (1.06,1.14)	251,650 (99.0)	2,630 (1.0)	0.82 (0.78, 0.86)	0.89 (0.85, 0.93)
**E-values**^**†**^ **for OR (and for CI)**			1.62 (1.54)	1.43 (1.31)			1.74 (1.60)	1.50 (1.36)

Abbreviations: GDM = gestational diabetes mellitus; OR = odds ratio; CI = confidence intervals.

^*^ Model adjusted for birth year, maternal age at delivery, cohabitation, smoking, maternal country of birth, maternal education, epilepsy, hypertension, psychiatric conditions, other comorbidities related to folic acid use, antiepileptic medication, medication used to treat psychiatric conditions, methotrexate use, and glucocorticoids use

^**†**^ E-value represents the minimum strength of association needed between an unmeasured confounder and both the exposure and the outcome to fully explain away the exposure-outcome association

The E-values for the adjusted ORs and CIs in the main analyses were 1.43 (1.31) and 1.50 (1.36) for Norway and Sweden, respectively (**Table 3**). Results from the exposure misclassification bias analysis showed that the crude ORs in both the Norwegian and Swedish cohorts corrected for lower sensitivity and specificity move farther away from the null (**S3 Table in S1 File**). However, the exposure misclassification bias analysis may help to understand the extent of the potential bias introduced by lower sensitivity and specificity levels but cannot explain the differences in the direction of the associations between Swedish and Norwegian data.

For women with prescribed folic acid use (**Table 4**), there were higher odds for the development of GDM in both the Norwegian (adjusted OR = 1.33, 95%CI 1.15–1.53) and Swedish (adjusted OR = 1.56; 95%CI 1.41–1.74) cohorts. The E-values for these estimates and CIs were 1.99 (1.57) and 2.49 (2.17), respectively, indicating that a stronger association between a potential unmeasured confounder and the exposure and outcome is required to explain away the findings. When adjusted for BMI, the strength of the association slightly decreased (**S2 Table in S1 File**).

**Table 4:** Crude and adjusted odds ratios and 95% confidence intervals estimating the association between prescribed folic acid use and gestational diabetes in mothers from Norway and Sweden.

	Norway				Sweden			
	No GDM n (%)	GDM n (%)	Crude OR (95% CI)	Adjusted * OR (95% CI)	No GDM n (%)	GDM n (%)	Crude OR (95% CI)	Adjusted * OR (95% CI)
**No Folic acid use**	238,755 (97.1)	7,060 (2.9)	Reference	Reference	828,160 (98.7)	10,570 (1.3)	Reference	Reference
**Prescribed folic acid use**	6,619 (95.7)	294 (4.3)	1.50 (1.33, 1.70)	1.33 (1.15, 1.53)	19,371 (97.8)	436 (2.2)	1.76 (1.60, 1.95)	1.56 (1.41, 1.74)
**E-values** ^**†**^ **for OR (and for CI)**			2.37 (1.99)	1.99 (1.57)			2.92 (2.58)	2.49 (2.17)

Abbreviations: GDM = gestational diabetes mellitus; OR = odds ratio; CI = confidence intervals.

^*^ Model adjusted for birth year, maternal age at delivery, cohabitation, smoking, maternal country of birth, maternal education, epilepsy, hypertension, psychiatric conditions, other comorbidities related to folic acid use, antiepileptic medication, medication used to treat psychiatric conditions, methotrexate use, and glucocorticoids use.

^**†**^ E-value represents the minimum strength of association needed between an unmeasured confounder and both the exposure and the outcome to fully explain away the exposure-outcome association

## Discussion

In the two Nordic population-based cohorts, pregnant women who self-report use of folic acid had slightly higher odds of GDM compared to non-users in Norway, and slightly lower odds in Sweden. In both the Norwegian and Swedish cohorts, higher odds for GDM were found in women with prescribed folic acid versus non-users of folic acid.

The different directions of the association for the self-reported folic acid and GDM analyses between the Norwegian and Swedish cohort results were surprising since diet, lifestyle, socioeconomic and health factors, and folic acid use recommendations are similar between the populations in Norway and Sweden. Further, population health registers were used from both countries and analyses were conducted using a common protocol. However, possible sources behind the inconsistency may be related to exposure and outcome misclassification, specifically to differences in how information on folic acid use was collected and the differences in diagnostic criteria used to establish a diagnosis of GDM. In the Norwegian population, women are specifically asked by the midwife about folic acid supplementation during the first antenatal visit, while in the Swedish population, pregnant women in the same setting are asked to report any medication use. This may have resulted in a substantial proportion of women who used folic acid to be misclassified as non-users in the Swedish population. There is no validation of the self-reported folic acid use data in the Norwegian or Swedish Medical Birth Registers. However, other Swedish studies have reported that while only approximately 20–30% of women take folic acid during the pregnancy planning period, around 60% of pregnant women were using folic acid during their current pregnancy [17–19], therefore providing some evidence that there is likely to be a substantial amount of misclassification of users as non-users in the Swedish data used for this study. We speculate that the misclassification of self-reported folic acid use is non-differential with respect to the outcome, first because the exposure information is collected in early pregnancy before the screening and diagnosis of GDM (in mid- to late pregnancy), avoiding bias introduced by potential over-reporting amongst women with GDM diagnosis. The exposure misclassification bias analysis showed that the consequence of a lower sensitivity of the exposure (and therefore more women with folic acid use being inaccurately classified as not using folic acid) in the Swedish data may result in a lower OR for the association between self-reported folic acid use and GDM, but would not explain the difference in direction of the association between the two Nordic cohorts.

Further, the prevalence of diagnosed GDM was lower in Sweden than in Norway. During the study period, Sweden, compared to Norway, had a higher threshold for identifying hyperglycemia in pregnancy and therefore the sensitivity of the outcome measure is lower, resulting in non-differential misclassification of cases of GDM as not having GDM in the Swedish data, which may influence the direction of the association found [20].

The results between the two cohorts for the prescribed folic acid analysis were similar. As expected, women in our Norwegian and Swedish cohorts with prescribed folic acid dispensations had higher proportion of comorbidities such as epilepsy and use of antiepileptic medication, which is in line with the folic acid recommendations. Additionally, a notable proportion of women in this group were using methotrexate or had diagnoses often treated with methotrexate (e.g. rheumatoid arthritis, psoriasis, Crohn’s disease, immune deficiency, etc.) which may impair folate activity and therefore higher dose folic acid use is recommended [21]. Notably, many of these conditions are associated with the development of GDM and other pregnancy complications, and were therefore adjusted for in the analysis models.

### Strengths and limitations

This study included two large cohorts based on pregnancies in the entire population of two Nordic countries, which allowed us to have power to detect associations between common exposures and relatively common outcomes. Information on maternal folic acid levels were not available but reported folic acid use has been shown to correspond well with plasma folate levels in a sample of Swedish women [19] and similarly in Norway [22]. We also did not have information on the duration of folic acid supplement use nor precise information on dose amongst the self-reported users. Since pregnancies from women with dispensed prescriptions of higher dose folic acid (1–5 mg/day) were identified separately, we assume that the women with self-reported folic acid use were taking the recommended 0.4 mg supplementation, alone or in a prenatal multivitamin.

In this study we had limited ability to adjust for potential confounding variables, such as lifestyle factors, which are associated with folic acid use and GDM onset. It has been shown that women using folic acid around pregnancy have a mix of both protective and risk factors for GDM. In a Norwegian study, women who had used supplements regularly from 1 month before pregnancy throughout the first trimester were older, non-smokers, had higher income and education, pregnancies were more often planned, and had higher rates of infertility treatment and chronic diseases [23]. Whereas a Swedish study, found that education level and employment status were the most significant factors related to folic acid supplement use in pregnant women [19].

To assess the robustness of the findings in each of the cohorts, to potential unmeasured confounders, we have reported the E-values which quantify the strength of the association between a confounder and the exposure and outcome needed to explain away the findings [12]. For the results of the self-reported folic acid use, the association between a potential unmeasured confounder and folic acid and GDM was similar in the Norwegian and Swedish cohorts, and should correspond to an OR of at least 1.43 and 1.50, respectively. It is therefore possible that being able to adjust for a lifestyle factor such as diet or physical activity would lead to estimates closer to, and including, the null.

### Findings in context of previous studies

There are inconsistent results amongst previous studies investigating folic acid use and GDM onset, which are summarized in the **S4 Table in S1 File**. The inconsistency may be due to multiple factors including differences in prevalence of GDM in the study populations, diagnostic criteria for GDM, folic acid use definitions and measurements, clinical traditions surrounding folic acid use in women at higher risk for GDM, covariates accounted for in the analysis, and differences in the diet and lifestyle of the populations in which the studies are conducted.

The only two other observational studies which found an inverse association similar to the results in our Swedish cohort included a large study of 20,119 pregnancies in the US and an even larger study of 187,432 pregnancies from the city of Xiamen in China [8, 24].

In contrast to this US study, Li et al., (2019) reported that in a maternal health cohort study in China (n = 4353), in women using >0.8 mg of folic acid for more than 4 weeks during pregnancy the odds for GDM were higher compared to non-folic acid users (adjusted OR = 2.09 (95% CI 1.30, 3.36)). In women using the same amount for less than 4 weeks, or using 0.4 mg, there was no association with GDM [25]. For both these studies, it’s unknown if the women were using higher doses of folic acid due to comorbidities and co-medications.

Duration of use of folic acid supplementation appears to also have a significant role, as Cheng et al., (2019) and Huang et al., (2019) reported that women using 0.4 mg/day for more than 3 months pre-pregnancy and in early pregnancy had an higher risk for GDM compared to women with no use or use for less than 2 months, respectively [26, 27]. The blood concentration of folate at which metabolic effects are expected in pregnant women are unknown, however studies have shown that to achieve the desired red blood cell concentration of folate needed to prevent neural tube defects (≥ 906 nmol/L), supplementation with 0.4 mg of folic acid for 12 weeks is required [28]. A meta-analysis of observational studies which measured folate in maternal serum or red blood cells (which reflect short and long term exposure, respectively) reported a significant positive association between maternal high folate status and GDM [9].

The inconsistency in the association between folic acid use and diabetes also exists among studies in non-pregnant populations. For example, in a cohort of 7333 Korean adults, an inverse association between dietary folic acid intake and diabetes incidence has been reported among women [29]. However, other studies have reported a potential benefit of folic acid supplementation on insulin resistance and glycemic control, but no clear effect on the development of diabetes [30–33].

There are biological mechanisms which support folic acid being both a risk factor and a protective factor for GDM. For example, physiologically insufficient levels of folic acid leads to high homocysteine levels, which associates with metabolic disturbances including insulin resistance in animal models [4], as well as in humans with type 1 or 2 or gestational diabetes [34]. Homocysteine levels are also significantly elevated in women with GDM compared to women with normal glucose tolerance in the second trimester [35]. Hence, higher folic acid intake may keep homocysteine levels at physiological level. However, a randomized controlled trial testing the effect of lowering homocysteine levels with daily supplements including folic acid and other B vitamins found no reduction of risk for type 2 diabetes in high-risk women [30]. Further, high dose folic acid supplementation has shown to improve parameters of insulin resistance in rodent models via AMP activated kinase pathways [5], which are a major cellular regulator of lipid and glucose metabolism and the same pathways targeted by oral antidiabetic medications including metformin [36, 37]. However, high folic acid status has been shown to exaggerate the metabolic effects of vitamin B12 deficiency [38], and therefore may participate in the pathogenesis of GDM through worsening insulin resistance.

#### Unanswered questions and future research

Inconsistency in the results from the numerous studies conducted on this topic warrant future work on elucidating relevant mechanisms by which folic acid could alter GDM risk. Because folic acid is an inexpensive and safe supplement which would be easy to implement in the prevention of GDM, additional randomized controlled studies should be conducted in various study populations to obtain more definitive results without the influence of confounding factors. Future observational studies should aim to collect more precise information on the dose, duration, and time of use of folic acid to improve the accuracy of the estimated associations.

## Conclusion

While there was higher or lower odds for GDM amongst women self-reporting folic acid use in Norway and Sweden, respectively, these results are likely not of clinical relevance, and should not influence folic acid use recommendations. Similar to previous studies, results from two Nordic cohorts showed different directions for the association between self-reported folic acid use and GDM, yet results from the exposure misclassification bias analyses show that exposure misclassification are unlikely explanations for the differences, while differences in prevalence of use and residual confounding still remain a concern. Prescribed folic acid is of a higher dose than recommended and is typically prescribed to women with specific comorbidities and co-medications, which likely underlies the reported higher odds for GDM.

## Supporting information

S1 File Literature search, methods and results. (PDF)

S1 Checklist STROBE checklist. (PDF)
