# Supplementary material for: Association between folic acid use during pregnancy and gestational diabetes mellitus: Two population-based Nordic cohort studies
Source: PLoS One. 2022 Aug 11;17(8):e0272046. doi: 10.1371/journal.pone.0272046 (PMC9371283; doi:10.1371/journal.pone.0272046)

Supplementary Information – Literature Search, Methods, and Results

Association between folic acid use during pregnancy and gestational diabetes mellitus: two population-based Nordic cohort studies

Authors: Laura Pazzagli, Silvia Segovia Chacón, Christos Karampelias, Jacqueline M Cohen, Gabriella Bröms, Helle Kieler, Ingvild Odsbu, Randi Selmer, Olov Andersson, Carolyn E Cesta*

***Corresponding Author:**
Carolyn E Cesta, Eugeniahemmet T2, Department of Medicine Solna, Karolinska Institutet, 171 76 Stockholm, Sweden. carolyn.cesta@ki.se https://orcid.org/0000-0001-5759-9366

Contents

S1 Methods. Identifying maternal self-reported folic acid use in the Swedish Medical Birth Register

S1 Table. ICD and ATC codes used to identify covariates

S2 Table. BMI-adjusted odds ratio and 95% confidence intervals estimating the association between self-reported and prescribed folic acid use and gestational diabetes

S3 Table. Exposure misclassification bias analysis corrected odds ratios and 95% confidence intervals for the association between self-reported folic acid use and gestational diabetes

S4 Table. Summary of Previous Studies investigating the association between folic acid use in pregnancy and onset of gestational diabetes mellitus (GDM)

S2 Methods. Literature search strategy and data extraction method

S1 Figure. PRISMA Flowchart of retrieval and selection of studies

S1 Methods. Identifying maternal self-reported folic acid use in the Swedish Medical Birth Register

In Sweden, at the first antenatal care visit around 10-12 weeks gestation, the pregnant woman is asked about any medication use during pregnancy, and the information is recorded as free text in the patient's chart and then included in the Medical Birth Register since 1995. In some cases, the free text is converted into an ATC code by the register holder. Information about folic acid use was extracted from the free text variable based the following ATC codes, folic acid supplement names, and pregnancy-specific multivitamin supplement names:

A11A, A11AA, A11AA01, A11AVIT, A11E, A11EA, A11EA00, A11EA1A, A11EA1B, A11EA2A, A11EA2B, A11EA2H, B03AD03, B03AE02, B03BB01, B03BB51, B12F, B12FO, B12FOL, BEFOLAT, BIFOLAC, BIOFOL, BIOFOLI, BIOFOLL, FEMBION, FEMIBIO, FOLAT, FOLBIOL, FOLICA, FOLICAC, FOLIFER, FOLS, FOLSYRA, TRIOB, TRIOBE, TRIOBEE, ZIFOLET, biofol

S1 Table. ICD and ATC codes used to identify covariates

**Covariate**	**ICD codes recorded in the MBRs or ATC codes recorded in the PDRs in the six months before the start of pregnancy**
Epilepsy	ICD-10 G40, G41
Hypertension	ICD-10 O10, O11
Psychiatric disorders	ICD-10 F10-99
Antiepileptics	ATC N03A
Medications used to treat psychiatric conditions	ATC N05, N06
Other comorbidities related to folic acid use	ICD-10:<br>D80 Immune deficiency with predominant antibody defects<br>D81 Combined immunodeficiencies<br>D82 immunodeficiencies associated with other major defects<br>D83 Common variable immunodeficiency<br>D84 Other immunodeficiencies<br>K50 Crohn's disease<br>L40 Psoriasis<br>M05 Seropositive rheumatoid arthritis<br>M06 Other rheumatoid arthritis<br>M07 Joint disorders in psoriasis and inflammatory bowel disease<br>M08 Juvenile arthritis<br>M13.0 Unspecified polyarthritis<br>M30 Polyarteritis nodosa and related conditions<br>M31 Other necrotizing vasculitis<br>M32 Systemic lupus erythematosus [SLE]<br>M33 Dermatopolymyositis<br>M34 Systemic sclerosis<br>M35 Other systemic involvement of connective tissue<br>M45 Ankylosing spondylitis<br>M46.1 Sacroiliitis, not elsewhere classified<br>M46.8 Other specified inflammatory disorders of the spine<br>M94.1 Relapsing polychondritis
Methotrexate	ATC L04AX03
Glucocorticoids	ATC H02AB

Abbreviations: ATC - Anatomical Therapeutic Chemical Classification System; ICD-10 - International Classification of Diseases and Related Health Problems 10th Revision; MBRs - Medical Birth Registers; PDRs - Prescribed Drug Registers.

S2 Table. BMI-adjusted odds ratio and 95% confidence intervals estimating the association between self-reported and prescribed folic acid use and gestational diabetes

	Norway	Sweden
	BMI adjusted model [a]<br>OR (95% CI)	BMI adjusted model [a]<br>OR (95% CI)
**No Folic acid use**	Reference	Reference
**Self-reported folic acid use**	1.13 (1.09, 1.17)	0.92 (0.88, 0.96)
**E-values [b] for OR (and for CI)**	1.51 (1.40)	1.39 (1.25)
**Prescribed folic acid use**	1.30 (1.13, 1.50)	1.49 (1.3, 1.66)
**E-values [b] for OR (and for CI)**	1.92 (1.51)	2.34 (2.01)

Abbreviations: BMI = body mass index; GDM = gestational diabetes mellitus; OR = odds ratio; CI = confidence intervals.

[a] Model adjusted for early-pregnancy BMI, birth year, maternal age at delivery, cohabitation, smoking, maternal country of birth, education, epilepsy, hypertension, psychiatric conditions, other comorbidities related to folic acid use, antiepileptic medication, medication used to treat psychiatric conditions, methotrexate use, and glucocorticoids use.

[b] E-value represents the minimum strength of association needed between an unmeasured confounder and both the exposure and the outcome to fully explain away the exposure-outcome association

S3 Table. Exposure misclassification bias analysis corrected odds ratios and 95% confidence intervals for the association between self-reported folic acid use and gestational diabetes

	Norway	Sweden
**Observed crude OR (95%CI)**	1.17 (1.14, 1.20)	0.82 (0.79, 0.86)
**Assumed Sensitivity and Specificity**	**Corrected crude OR (95% CI)**	**Corrected crude OR (95% CI)**
85 %, 85%	1.33 (1.26, 1.40)	0.57 (0.49, 0.66)
90 %, 90%	1.25 (1.20, 1.30)	0.71 (0.66, 0.77)
95 %, 95%	1.20 (1.16, 1.24)	0.78 (0.74, 0.83)
99 %, 99%	1.17 (1.14, 1.21)	0.82 (0.78, 0.85)

S4 Table. Summary of Previous Studies investigating the association between folic acid use in pregnancy and onset of gestational diabetes mellitus (GDM)

**Author Country (Year)**	**Study population**	**Folic acid use ascertainment**	**Covariates**	**Adjusted risk estimate for GDM**
**Observational Cohort Studies**				
**Chen<br>China<br>(2021)<br>[1]**	1058 pregnancies from the Shanghai Preconception Cohort Study	Self-reported folic acid supplementation in early pregnancy.	Models adjusted for:<br>age, preconception BMI, family history of diabetes, smoking exposure, and alcohol use status.	ORs of **GDM** according to:<br>daily **folic acid supplementation** (0.4 or 0.8 mg) in early pregnancy<br>OR = 1.73 (95% CI 1.19–2.53)
**Jankovic-Karasoulos<br>Australia<br>(2021)<br>[2]**	3196 singleton pregnancies from nulliparous women enrolled in the Adelaide–Auckland SCOPE cohort	Folic acid supplementation at 15 ± 1 weeks of gestation, data collected by research midwife interview.	N/A	Proportion of women who developed **GDM** by folic acid supplementation status:<br><br>No folic acid supplementation = 3.1%<br><0.8 mg folic acid supplementation = 3.4%<br>≥0.8 mg folic acid supplementation = 3.8%<br>*P* value = 0.7470
**Zhao<br>China<br>(2021)<br>[3]**	187,432 pregnancies from 10 “AAA” hospitals in Xiamen from 2008 and 2018.	Self-reported pre-pregnancy intake of folic acid at the 1st antenatal visit.	Models adjusted for:<br>age, smoking, alcohol use, education, age at menarche, maternal family histories of diabetes mellitus and hypertension, and the presence of hemopathy, epilepsy, hyperthyroidism, cardiovascular/liver/kidney and lung diseases.	ORs of **GDM** according to **pre-pregnancy intake of folic acid** (yes *vs* no)<br>OR = 0.73 (95% CI 0.69–0.79)
**Cheng<br>China<br>(2019)<br>[4]**	950 singleton pregnancies from women enrolled in the cohort study during 2015 in Changsha.	Duration of folic acid supplementation (0.4 mg/day)	Models adjusted for:<br>Maternal age, education, average monthly household income, postpartum BMI, parity, passive smoking, family history of diabetes, iron supplementation, multivitamin supplementation, gestational dietary intake, and alcohol use.	RRs of **GDM** for **folic acid supplementation** for ≥3 months compared to use for <3 months”<br><br>≥3 months pre-pregnancy: Adjusted RR: 1.72 (95% CI 1.17, 2.53)<br>≥3 months during pregnancy: Adjusted RR: 1.16 (95% CI 0.75, 1.81)<br><br>Note: non-folic acid users included in the <3 months group.

**Huang China (2019) [5]**	326 pregnancies enrolled into the cohort at 16-18 weeks of gestation between August 2014 and April 2015 in Hefei, Anhui Province.	Duration of folic acid supplementation (93.5% of participants reported taking 0.4 mg/day, with range of 0.14 to 1.2 mg/day)	Models adjusted for:<br>Age, pre-pregnancy BMI, education level, parity, passive smoking, monthly income, energy and overall nutrients intake, TG, HDL-c, LDL-c concentrations.	ORs of **GDM** according to tertiles of **duration of folic acid supplementation**:<br><br>Non-folic acid users OR = 1.53 (95% CI 0.24, 9.87)<br>1-60 days reference<br>61-90 days OR = 1.25 (95% CI 0.32, 4.85)<br>91-360 days OR = 3.45 (95% CI 1.01, 11.8)
**Li USA (2019) [6]**	20,199 pregnancies to 14,553 women between 1991 and 2001 participating in the Nurses' Health Study II.	Dietary, supplemental, and total folic acid intake	Models adjusted for:<br>age, race, parity, family history of diabetes, pre-pregnancy BMI, cigarette smoking, alcohol use, physical activity, total energy intake, glycemic load, intake of saturated fat, total fiber, heme iron.	RRs of **GDM** according **to pre-pregnancy folate intake.**<br>**Total folate intake (mg/day)**<br>Q1(0.081–0.293) reference<br>Q2(0.294–0.422) RR= 1.01 (95% CI 0.84, 1.21)<br>Q3(0.423–0.697) RR= 0.81 (95% CI 0.67, 0.99)<br>Q4(0.698–2.770) RR= 0.81 (95% CI 0.66, 0.98)<br>**Adequate total folate intake**<br>≥0.400 mg/day RR= 0.83 (95% CI 0.72, 0.95)<br>**Supplemental folate intake (mg/day)**<br>0 reference<br>0.001–0.399 RR = 0.83 (95% CI 0.71, 0.98)<br>0.400–0.599 RR = 0.77 (95% CI 0.64, 0.93)<br>≥0.600 RR = 0.70 (95% CI 0.52, 0.94)
**Li China (2019) [7]**	4,353 singleton pregnancies from women participating in the Tongji Maternal and Child Health Cohort (TMCHC).	Dose and duration of supplemental folic acid	Models adjusted for:<br>Age, education, employment, monthly income, ethnicity, primiparity, pre-pregnancy BMI, smoking, drinking, family history of diabetes, other supplement use, and weight gain at OGTT.	ORs for **GDM** according to **folic acid supplement use.**<br>**Reference group:** non-users, daily dose <0.4 mg, and/or duration <4 weeks<br>**0.4–0.8 mg/day for less than 4 weeks:**<br>Crude OR = 1.23 (95% CI 0.84, 1.80)<br>**0.4–0.8 mg/day for more than 4 weeks:**<br>Crude OR = 1.23 (95% CI 0.78, 1.94)<br>**>0.8 mg/day for less than 4 weeks:**<br>Crude OR = 1.37 (95% CI 0.93, 2.01)<br>**>0.8 mg/day for more than 4 weeks:**<br>Crude OR = 2.36 (95% CI 1.51, 3.69)<br>Adjusted OR = 2.09 (95% CI 1.30, 3.36)
**Zhu China (2016) [8]**	1,938 pregnancies from women recruited during the 1st or 2nd trimester into the population-based	Folic acid supplementation<br>No dose reported	Not reported	OR for **GDM** according to **folic acid supplement consumption in the first trimester**:<br>Adjusted OR = 2.25 (95% CI 1.35, 3.76),<br>Reference: women who never used vitamin supplements<br>OR for GDM according to women with a **pre-pregnancy BMI ≥25 kg/m2** and taking folic acid **supplements daily in the first trimester**.

	China-Anhui Birth Cohort Study (C-ABCS) between May 2013 and September 2014.			OR = 5.63 (95% CI 2.77, 11.46)<br>Reference: with women with a pre-pregnancy BMI <25 kg/m2 and not taking any FA supplements.
**Intervention Studies**				
**Li**<br>**China**<br>**(2015)**<br>**[9]**	2,928 pregnancies in the intervention arm (folic acid supplementation) and 4,884 pregnancies in the control group, recruited 2012-2013 from the Jiaodong region in Shandong.	**Intervention group:** Folic acid supplementation with doses (0.4 or 0.8 mg) assigned by the ranked folic acid metabolism ability determined by allele polymorphism frequencies of specific of genes involved in folic acid metabolism<br>**Control group:** Pregnant women who did not receive folate supplementation in the same period at the same hospital.	Not reported<br><br>**Exclusion criteria:** Women who smoked, drank alcohol, used prescription medications during the perinatal period and had a diagnosis of a chronic diseases.	Proportion of **GDM** diagnosed in intervention and control group<br><br>intervention = 8 (0.27%)<br>control = 158 (3.24%)<br>p<0.050

Abbreviations: BMI: Body mass index, **CI**: Confidence intervals; **GDM**: Gestational diabetes mellitus; **OR**: Odds ratio; **RR**: Relative risk.

Table S4 Summary:

Nine studies met the inclusion criteria for the literature review. Search results are presented in **Supplementary Figure 1. Table S4** reports the study details and results for the included studies. There were 8 observational cohort studies and 1 intervention study. The studies varied in their primary exposure definition: Folic acid use pre-pregnancy (n=1) or in early pregnancy (n=4), specific doses of folic acid use (n=2), and/or duration of folic acid use (n=3). The majority of the studies adjusted their analysis models for maternal age, parity, BMI, education and other markers of social economic status, smoking, while some also included information about family history of diabetes, and dietary intake. Overall, 5 observational studies from China reported an increased risk with folic acid use before or during pregnancy, and when measured, longer duration of use and higher doses showed stronger associations.[1, 4, 5, 7, 8] One study from Australia found no increased prevalence of GDM amongst folic acid users.[2] The two largest cohort studies, conducted in the US and in China, were the only observational studies to report a protective association between folic acid use and GDM.[3, 6] The intervention study reported a lower proportion of GDM in the folic acid intervention group which received a dose related to their rank metabolism ability of folic acid compared to the control group which did not receive folic acid supplements.[9]

Table References

1. Chen X, Zhang Y, Chen H, Jiang Y, Wang Y, Wang D, et al. Association of Maternal Folate and Vitamin B(12) in Early Pregnancy With Gestational Diabetes Mellitus: A Prospective Cohort Study. Diabetes care. 2021;44(1):217-23. Epub 2020/11/08. doi: 10.2337/dc20-1607. PubMed PMID: 33158950; PubMed Central PMCID: PMCPMC7783943.
2. Jankovic-Karasoulos T, Furness DL, Leemaqz SY, Dekker GA, Grzeskowiak LE, Grieger JA, et al. Maternal folate, one-carbon metabolism and pregnancy outcomes. Maternal &

child nutrition. 2021;17(1):e13064. Epub 2020/07/29. doi: 10.1111/mcn.13064. PubMed PMID: 32720760; PubMed Central PMCID: PMCPMC7729528.
3. Zhao M, Yang S, Hung TC, Zheng W, Su X. Association of pre- and early-pregnancy factors with the risk for gestational diabetes mellitus in a large Chinese population. Sci Rep. 2021;11(1):7335. Epub 2021/04/03. doi: 10.1038/s41598-021-86818-7. PubMed PMID: 33795771; PubMed Central PMCID: PMCPMC8016847.
4. Cheng G, Sha T, Gao X, He Q, Wu X, Tian Q, et al. The Associations between the Duration of Folic Acid Supplementation, Gestational Diabetes Mellitus, and Adverse Birth Outcomes based on a Birth Cohort. Int J Environ Res Public Health. 2019;16(22). Epub 2019/11/17. doi: 10.3390/ijerph16224511. PubMed PMID: 31731641; PubMed Central PMCID: PMCPMC6888242.
5. Huang L, Yu X, Li L, Chen Y, Yang Y, Yang Y, et al. Duration of periconceptional folic acid supplementation and risk of gestational diabetes mellitus. Asia Pac J Clin Nutr. 2019;28(2):321-9. Epub 2019/06/14. doi: 10.6133/apjcn.201906_28(2).0014. PubMed PMID: 31192561.
6. Li M, Li S, Chavarro JE, Gaskins AJ, Ley SH, Hinkle SN, et al. Prepregnancy Habitual Intakes of Total, Supplemental, and Food Folate and Risk of Gestational Diabetes Mellitus: A Prospective Cohort Study. Diabetes care. 2019;42(6):1034-41. Epub 2019/04/24. doi: 10.2337/dc18-2198. PubMed PMID: 31010874; PubMed Central PMCID: PMCPMC6609948.
7. Li Q, Zhang Y, Huang L, Zhong C, Chen R, Zhou X, et al. High-Dose Folic Acid Supplement Use From Prepregnancy Through Midpregnancy Is Associated With Increased Risk of Gestational Diabetes Mellitus: A Prospective Cohort Study. Diabetes care. 2019;42(7):e113-e5. Epub 2019/05/12. doi: 10.2337/dc18-2572. PubMed PMID: 31076420.
8. Zhu B, Ge X, Huang K, Mao L, Yan S, Xu Y, et al. Folic Acid Supplement Intake in Early Pregnancy Increases Risk of Gestational Diabetes Mellitus: Evidence From a Prospective Cohort Study. Diabetes care. 2016;39(3):e36-7. Epub 2016/01/30. doi: 10.2337/dc15-2389. PubMed PMID: 26822325.
9. Li X, Jiang J, Xu M, Xu M, Yang Y, Lu W, et al. Individualized supplementation of folic acid according to polymorphisms of methylenetetrahydrofolate reductase (MTHFR), methionine synthase reductase (MTRR) reduced pregnant complications. Gynecol Obstet Invest. 2015;79(2):107-12. Epub 2015/01/31. doi: 10.1159/000367656. PubMed PMID: 25634728.

S2 Methods. Literature review search strategy and data extraction method

A comprehensive search of the literature was conducted to identify relevant studies investigating the association between folic acid use before and/or during pregnancy in humans. Observational and interventional studies were included with no early date limit and ending in April 2021.

The search was conducted in the following databases: Medline (OVID), Embase.com and Web of Science (Clarivate) in August 2019 and updated in April 2021. The following MeSH-terms were used in the Medline (OVID) search: ‘Diabetes, Gestational’ and ‘Folic Acid’. The MeSH-terms were adapted in accordance to the corresponding vocabulary in Embase. Each search concept was complemented with relevant free-text terms. The free-text terms were truncated and/or combined with proximity operators. The search was restricted to articles in English and databases were searched from inception. In addition to the search of electronic databases, reference lists of relevant articles were also reviewed to find potentially relevant studies that had not been identified by the database search strategy.

One reviewer evaluated the retrieved titles and abstracts. Articles identified were obtained for full-text review. Articles were excluded when the exposure (folic acid supplementation) or outcome (GDM) of interest was not included or if the study did not include human subjects. Only primary research studies were included. For each of the final 9 studies, the reviewer extracted the following information: author, study year, country, study design, study population, ascertainment of folate supplementation, and risk estimate for GDM. A second reviewer confirmed the accuracy of the extracted information.

S1 Figure. PRISMA Flowchart of retrieval and selection of studies

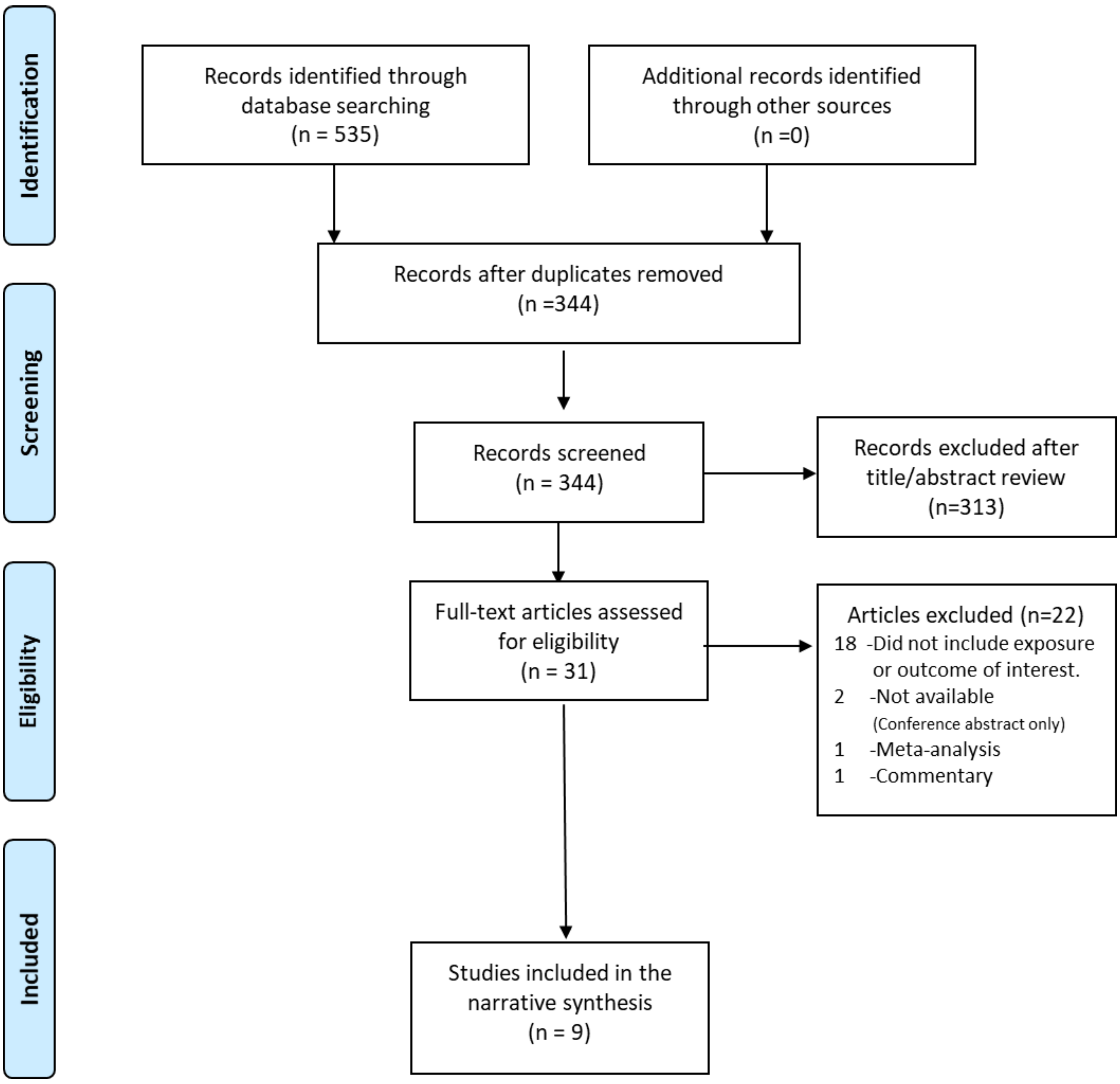

Supplement: S1 File — (PDF) [file pone.0272046.s001.pdf]
